# Cytokines in Allergic Conjunctivitis: Unraveling Their Pathophysiological Roles

**DOI:** 10.3390/life14030350

**Published:** 2024-03-07

**Authors:** DeGaulle I. Chigbu, Nicholas J. Karbach, Sampson L. Abu, Navpreet K. Hehar

**Affiliations:** Pennsylvania College of Optometry at Salus University, Elkins Park, PA 19027, USA; dchigbu@salus.edu (D.I.C.); nkarba@salus.edu (N.J.K.); sla0001@salus.edu (S.L.A.)

**Keywords:** Th2 cells, IL-4, IL-5, IL-6, IL-9, IL-13, IL-25, IL-31, mast cells, pathophysiology

## Abstract

Allergic conjunctivitis is one of the common immune hypersensitivity disorders that affect the ocular system. The clinical manifestations of this condition exhibit variability contingent upon environmental factors, seasonal dynamics, and genetic predisposition. While our comprehension of the pathophysiological engagement of immune and nonimmune cells in the conjunctiva has progressed, the same cannot be asserted for the cytokines mediating this inflammatory cascade. In this review, we proffer a comprehensive description of interleukins 4 (IL-4), IL-5, IL-6, IL-9, IL-13, IL-25, IL-31, and IL-33, as well as thymic stromal lymphopoietin (TSLP), elucidating their pathophysiological roles in mediating the allergic immune responses on the ocular surface. Delving into the nuanced functions of these cytokines holds promise for the exploration of innovative therapeutic modalities aimed at managing allergic conjunctivitis.

## 1. Introduction

Allergic disorders of the conjunctiva are IgE- or non-IgE-mediated immunological responses triggered by allergens exposed to the ocular surface. These encompass various clinical forms including seasonal allergic conjunctivitis (SAC), perennial allergic conjunctivitis (PAC), atopic keratoconjunctivitis (AKC), and vernal keratoconjunctivitis (VKC), where the former two constitute the majority of cases [1,2,3,4]. Approximately 20% of the world population have at least one clinical expression associated with allergies, of which over 40% of these individuals experience ocular involvement [5,6]. The prevalence of ocular allergies continues to increase globally, with a wide variation in geographic distribution that is influenced by environmental factors, allergen species, and differences in ethnicities [7]. It is general knowledge that individuals with a history of atopy have a genetic predisposition to develop allergic diseases such as allergic conjunctivitis. As an ocular surface disorder characterized by hypersensitivity immune reaction, allergic conjunctivitis usually presents bilaterally with pruritis as the hallmark feature that can be mild or debilitating [8,9]. Additional cardinal features of this condition include conjunctival hyperemia, tearing, and conjunctival chemosis. The immune response to an allergen on the ocular surface causes clinically significant symptomology. Whereas SAC and PAC are the milder forms of the disease, AKC and VKC can have deleterious effects on the ocular surface and potentially compromise vision. Allergic ocular surface diseases are therefore of public health concern given the potential adverse impact on a person’s quality of life. Allergic conjunctivitis involves innate and adaptive immune responses [10]. Macrophages, dendritic cells, and mast cells participate in the innate immune response to allergens that cross the conjunctival epithelium. During the innate immune response to allergens, dendritic cells and mast cells serve as a link between innate and adaptive immune responses [11,12]. Th2 and Th9 cells play major roles in the adaptive immune response in the conjunctiva [13]. There is a substantial body of evidence in the conjunctival allergen challenge (CAC) model supporting the role of Th2 cytokines in the pathophysiology of allergic conjunctivitis [14]. Th2 cells secrete IL-4, IL-5, IL-6, IL-9, IL-10, and IL-13, whereas Th9 cells secrete IL-9 and IL-10 [15]. Additional cytokines produced by Th2 cells include IL-25 (IL-17E) [16] and IL-31 [17]. IL-33 is another cytokine that mediates the type 2 immune response in the conjunctiva [18]. The cytokines secreted by Th2 and Th9 cells lead to the development of the pathophysiological changes observed in the ocular surface of individuals with allergic conjunctivitis. Because of the role of these cytokines in the pathophysiology of type 2 immune responses in allergic conjunctivitis, targeting these cytokines that mediate type 2 immune responses is a promising immunotherapeutic modality in allergic disease of the eye [14,15].

The conjunctiva is an immunologic tissue, and it is a component of the eye-associated lymphoid tissue. The eye-associated lymphoid tissue. Bronchus-associated lymphoid tissue and nasal-associated lymphoid tissue are components of the mucosal-associated lymphoid tissue (MALT) [19]. The ocular mucosal immune system has similar characteristics to the mucosal immune system of the lungs [20]. Additionally, components of the MALT have a lot of similarities in their mechanism of type-2 immune-mediated responses [21]. The immunopathophysiology of allergic conjunctivitis is similar to that of asthma and allergic rhinitis because Th2-derived cytokines, Th9-derived cytokine, IL-33, and TSLP induce the type 2 allergic immune-mediated inflammatory processes in these allergic diseases [19]. The rationale for selecting IL-4, IL-5, IL-6, IL-9, IL-13, IL-25, IL-31, IL-33, and TSLP is that these cytokines induce the type 2 allergic immune responses associated with atopic dermatitis, asthma, allergic rhinitis, and allergic conjunctivitis [22]. Additionally, the majority of the resident immune cells and other nonimmune cells in the conjunctiva express receptors for Th2- and Th9-derived cytokines. In a nutshell, the pathophysiology of allergic inflammation elsewhere in the body is not unique from the allergic reaction on the ocular surface.

In this comprehensive review, we thoroughly examined the peer-reviewed literature elucidating the intricate role of cytokines on allergic inflammation, with a keen interest in ocular allergy. Articles were sourced from prominent databases such as PubMed, Scopus, and Web of Science. To obtain the most recent available data, but ensure a comprehensive assessment of existing information, the search was restricted to 2000–2024. The keywords used for the search included cytokines, ocular allergy, allergic conjunctivitis, and other related terms. We prioritized articles presenting original research, clinical trials, and reviews that contribute substantively to the current understanding of cytokine-mediated mechanisms in ocular allergy. Exclusion criteria involve studies lacking relevance to cytokine involvement in ocular allergy, non-English publications, and those with insufficient methodological rigor. This meticulous selection process ensured a robust foundation for synthesizing the existing knowledge and advancing our comprehension of the involvement of cytokines in allergy of the ocular surface. This article provides a comprehensive review of the pathophysiological role of cytokines produced by both Th2 and Th9 cells in inducing the clinical expression of allergic conjunctivitis.

## 2. The Conjunctiva

The conjunctiva is a mucous membrane that covers most of the anterior surface of the eye and the posterior surface of the upper and lower eyelids. It can be divided into three regions: the palpebral conjunctiva, the bulbar conjunctiva, and the conjunctival fornix. The conjunctiva is composed of a surface layer of epithelial cells that are joined together by tight junctions and form a barrier to the external environment [23,24]. Conjunctival epithelial cells secrete membrane-bound mucins that extend from the apical membrane surface, forming a glycocalyx that adheres the mucin layer to the epithelial cells [25]. Goblet cells are unicellular apocrine glands that are present in the conjunctival epithelium. These cells secrete the highly glycosylated, hydrophilic glycoproteins that make up the mucin layer of the tear film [26]. The conjunctival stroma consists largely of collagen fibrils and fibroblasts interlaced with blood vessels, lymphatic vessels, and immune cells [11,23,27,28]. The blood supply for the conjunctiva comes primarily from the arcades of the lacrimal branch of the ophthalmic artery, which supplies the upper and lower lids. The venous drainage follows the path of the arterial supply and leads to the ophthalmic vein [24,29]. The conjunctiva contains a lymphatic drainage network with collector channels throughout the stroma draining into the preauricular, submandibular, and parotid lymph nodes [30,31]. Sensory innervation for the conjunctiva is provided by the supraorbital nerve, supratrochlear nerve, infraorbital nerve, infratrochlear nerve, and lacrimal nerve [24]. The conjunctiva participates in innate and adaptive immune responses [11,28]. Conjunctival epithelial cells play a role in receiving and sending immune signals. Leukocytes are found scattered throughout the healthy conjunctival epithelium [32,33,34]. The most common cell types in the conjunctival epithelium are CD8^+^T cells [33] and Langerhans cells [35,36,37]. T cells, IgA-secreting plasma cells, B cells, macrophages, dendritic cells, and mast cells are also found in the stroma [24,33,38,39]. The stromal T cell population is balanced between CD8^+^T cells and CD4^+^T cells [33,40]. Fibroblasts in the conjunctival stroma facilitate multiple aspects of immunity. The extracellular matrix (ECM) is produced by fibroblast [41]. Immune and nonimmune cells in the conjunctiva collectively orchestrate allergic inflammatory responses in this mucosal tissue. Of note, the roles of epithelial cells, fibroblast, mast cells, and dendritic cells are described. Epithelial cells of the ocular surface can participate in the pathogenesis of allergic conjunctivitis because of the expression of receptors for cytokines such as IL-9 and IL-33 [42,43]. Additionally, conjunctival epithelial cells can function as a mediator of allergen-induced conjunctival inflammation because activated conjunctival epithelial cells express cytokines and chemokines that mediate type 2 allergic immune responses [43,44,45,46]. Conjunctival fibroblasts express type I and type II IL-4 receptors, and binding of IL-4 and IL-13 to their receptors on conjunctival fibroblasts can induce conjunctival fibroproliferative changes [47,48,49]. Mast cells are usually located in the subepithelial layer of the conjunctiva. Tryptase and chymase are neutral proteases secreted by degranulated mast cells and are the basis for classifying mast cells. Mast cells that contain tryptase are mucosal mast cells located in the mucosa of the nose and lungs. Connective tissue mast cells contain tryptase and chymase, and they are usually located in the conjunctiva and skin [50]. Activated mast cells can release histamine, tryptase, lipid mediators, chemokines, and cytokines [51,52,53].

## 3. The Allergic Immune Response in the Conjunctiva

**Figure 1:**
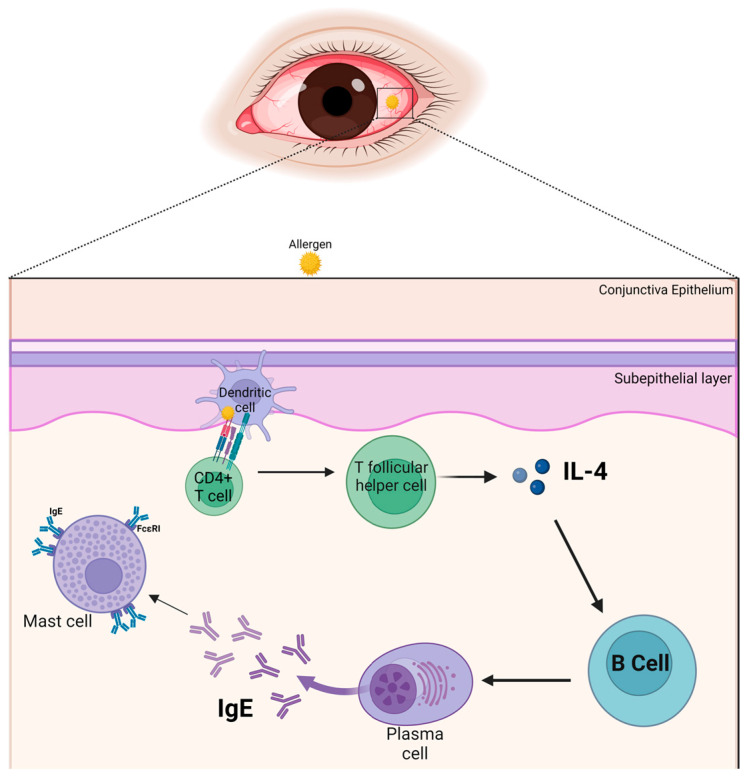
Priming of the conjunctival mast cells: Allergens on the conjunctiva are engulfed by dendritic cells and subsequently processed and presented to naïve CD4^+^T cells in the regional secondary lymphoid tissues. The allergen-specific CD4^+^T cell undergoes proliferation and differentiation into IL-4-secreting effector Follicular T helper cells. IL-4 drives the clonal expansion and differentiation of allergen-specific B cells into IgE-secreting plasma cells. The IgE released by the plasma cells binds to FcεRI on mast cells to render the conjunctival mast cells primed. Created with biorender.com.

The immunopathogenesis of allergic conjunctivitis is predominantly type I hypersensitivity immune reactions. Allergens on the conjunctiva such as pollen, an outdoor allergen, can trigger a type 1 hypersensitivity immune response in the conjunctiva. Allergens secrete protease that activates the protease-activated receptor-2 (PAR-2) in the epithelial cells [54]. Proteases secreted by pollen can breakdown the epithelial tight- and adherens junctions to facilitate the access of pollen to dendritic cells in the subepithelial layer to promote the sensitization phase of the allergic immune response or activation phase of the allergic immune response [55,56,57]. Furthermore, house dust mite is an indoor allergen that possesses protease activity, and the proteolytic activity of these allergens can disrupt the barrier function of the epithelial layer to facilitate the access of the allergen to antigen-presenting cells resident in the subepithelial layer of the conjunctiva to initiate the sensitization phase of the allergic immune response associated with allergic conjunctivitis [58,59,60]. Allergens in the subepithelial layer of the conjunctiva initiate a cascade of events during the sensitization phase of the allergic immune response, in which, allergens are engulfed by dendritic cells and subsequently processed and presented to naïve CD4^+^T cells in the regional secondary lymphoid tissues. The allergen-specific CD4^+^T cell undergoes proliferation and differentiation into IL-4-secreting effector CD4^+^T helper cells. IL-4 released by CD4^+^T helper cells drives the clonal expansion and differentiation of allergen-specific B cells into immunoglobulin E (IgE)--secreting plasma cells. The IgE released by the plasma cells binds to Fc Epsilon receptor I (FcεRI) on mast cells to render the conjunctival mast cells primed (Figure 1) [61,62]. Re-exposure of the conjunctiva to allergens triggers the activation phase of the allergic immune response, in which allergens use their proteolytic capabilities to break through the tight and adherence junctions that link the individual epithelial cells of the conjunctiva. Once inside the subepithelial layer of the conjunctiva, allergens bind to IgE attached to FcεRI on primed mast cells in the subepithelial layer of the conjunctiva. This interaction induces the crosslinking of these IgE-FcεRI on primed mast cells leading to activation and degranulation of mast cells to release mediators such as histamine, tryptase, lipid mediators, chemokines, and cytokines [58,63]. The activation phase of the allergic immune response results in acute clinical symptoms in all forms of the condition includes itching, redness, tearing, and swelling [5,8,9]. During the late phase of the allergic immune response, chemokines (CCL5) and cytokines (IL-4, IL-5, IL-9, and IL-13) are released during this phase of the allergic immune response. Cellular infiltration of eosinophils and Th2 cells to the site of allergic conjunctival inflammation in response to CCL5 is usually observed in perennial allergic conjunctivitis. A combination of preformed and newly formed mediators of allergic inflammation drives the pathophysiological changes that are observed on the ocular surface of individuals with allergic conjunctivitis. Thus, cytokines, along with other mediators of allergic inflammation, are responsible for the pathophysiological changes and the clinical expressions of the allergic inflammation of the conjunctiva [49,64].

## 4. Cytokines and Their Pathophysiological Roles

### 4.1. Interleukin-4

Interleukin-4 (IL-4) is a pleiotropic cytokine secreted by Th2 cells, mast cells, eosinophils, and basophils [65,66]. It is a glycoprotein with a four-helix bundled structure. As a pro-inflammatory cytokine, IL-4 is one of the only two cytokines that bind to the receptor, IL-4R [67]. Type I IL-4 receptor (IL-4R) is a heterodimeric receptor complex that consists of IL-4R alpha (CD124) and IL-2Rγ chain (CD132) [68,69]. Type I IL-4R is expressed on monocytes, macrophages, fibroblasts, T cells, and B cells, whereas type II IL-4R is expressed on monocytes, macrophages, smooth muscle cells, fibroblasts, and epithelial cells [54]. It is important to note that IL-4R alpha is expressed on epithelial cells of the cornea [70] and conjunctiva [71] (Table 1).

**Figure 2:**
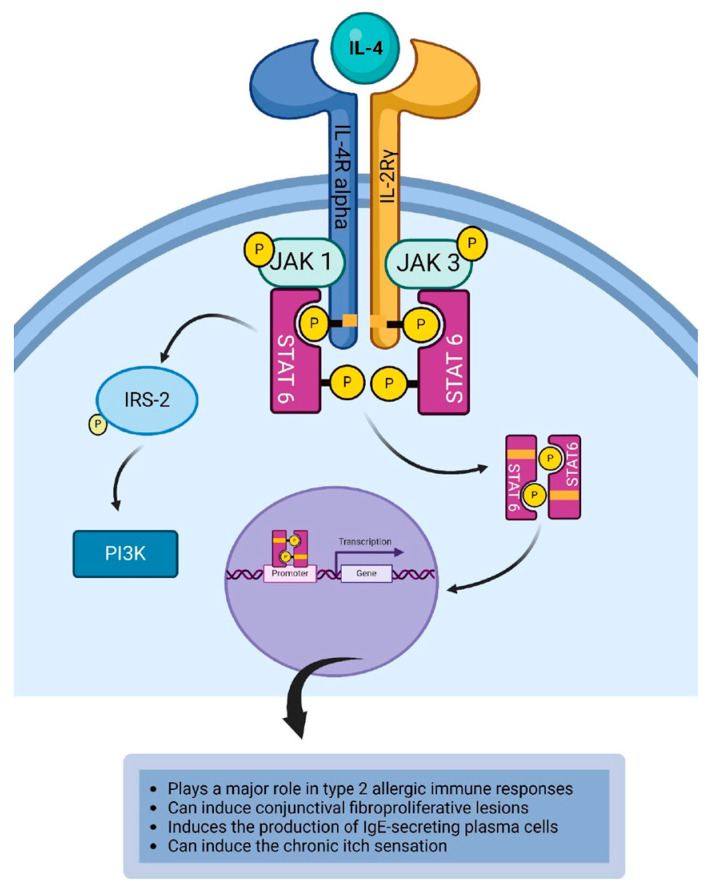
IL-4 binds to type I IL-4R to induce the JAK1/JAK3/STAT6 signaling cascade that results in the clinical manifestations of type 2 allergic immune response at the conjunctiva. Created with biorender.com.

IL-4 binds to type I IL-4R to induce the signaling cascade that results in the dimerization of the receptor. This brings Janus kinase 1 (JAK1) and JAK3 into proximity to phosphorylate each other. JAK1 is associated with the IL-4R alpha chain and JAK3 is associated with IL-2Rγ chain. The phosphorylated JAKs activate the tyrosine residues on the cytoplasmic domain of the IL-4R to create a phosphotyrosine docking site for signal transducer and activator of transcription 6 (STAT6) and Insulin Receptor Substrate-2 (IRS-2) [68,69,114,115,116]. IRS-2 is recruited to the IL-4R alpha, and it becomes phosphorylated by JAK1. The phosphorylated IRS-2 binds to phosphatidylinositol 3-kinase [65,114,115,117]. STAT6 is recruited to the IL-4R alpha, where it binds to JAKs. This results in the phosphorylation of STAT6 creating phosphorylated STAT homodimers, which subsequently dissociate from the cytoplasmic domain of the receptor to relocate to the nucleus and bind to the STAT-binding site on the DNA to initiate or regulate the transcription of IL-4 responsive genes that mediate the response of the cytokine (Figure 2) [118,119]. IL-4 and IL-13 can directly stimulate sensory neurons to induce the chronic itch sensation via the IL-4R alpha-JAK1 pathway [120]. Fukuda et al. [121] demonstrated that the expression of IL-4R (CD124) on corneal fibroblasts, and the activation of IL-4R expressed on corneal fibroblasts can lead to the secretion of CCL11 and CCL17. CCL11 induces the chemotaxis of eosinophils, mast cells, and Th2 cells, whereas CCL17 recruits Th2 cells [70,121]. As such, the interaction of IL-4 or IL-13 with their receptors expressed on corneal fibroblasts can contribute to the exacerbation of allergic inflammation of the ocular surface [121]. IL-4 modulates the differentiation of B cells [122,123,124]. It induces B cells to switch class and stimulates the production of IgE antibodies, a chief instigator of Type I hypersensitivity immune reaction. In allergic immune responses involving the conjunctiva, IgE binds to the receptors expressed by resident mast cells in the conjunctiva. This interaction culminates in the priming of the conjunctival mast cells, and subsequent allergen-induced crosslinking of these IgE bound to FcεRI on the primed mast cells causes degranulation of mast cells and release of histamine [125]. Conjunctival hyperemia and chemosis are cardinal signs of allergic conjunctivitis. During the activation phase of the allergic reaction, IL-4 binds to its receptors expressed on conjunctival vascular endothelial cells. This IL-4/IL-4R interaction can induce the upregulation of vascular cell adhesion molecule 1 (VCAM-1) on endothelial cells of the conjunctival vessels, which culminates in vasodilation and vasopermeability observed as conjunctival hyperemia and conjunctival chemosis, respectively [126,127]. IL-4 is responsible for activating conjunctival fibroblasts to undergo proliferation, which presents clinically as papillae on the palpebral conjunctiva in allergic conjunctivitis (Table 2) [128]. 

### 4.2. Intereukin-5

**Figure 3:**
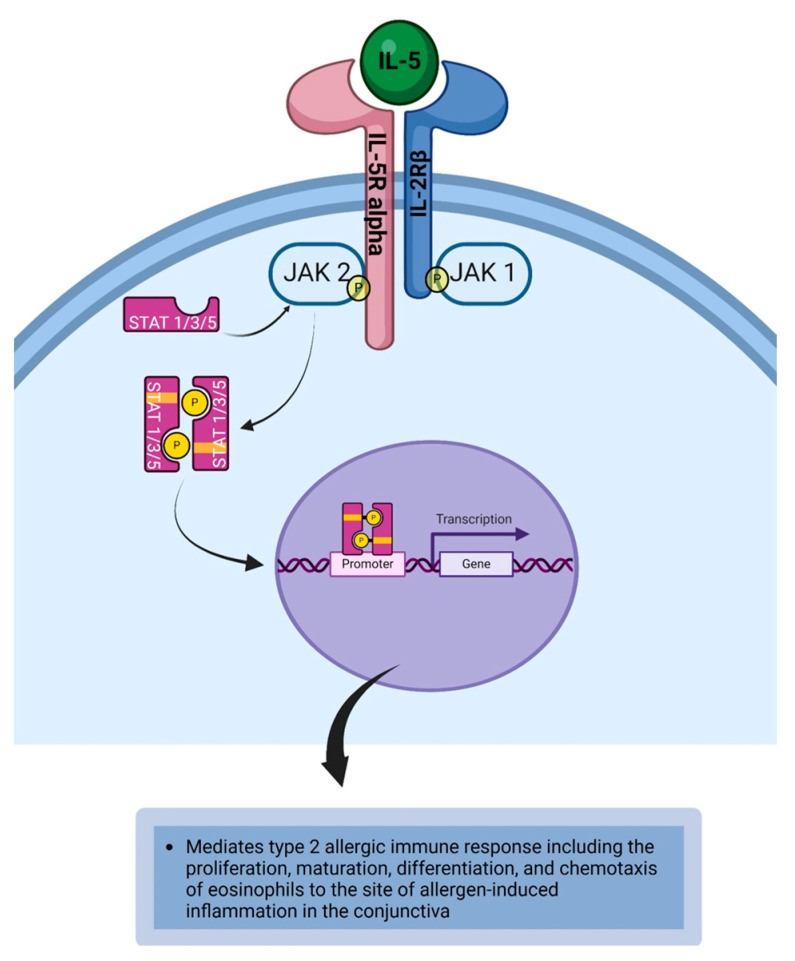
IL-5 binds to IL-5R to initiate the JAK1/JAK2/STAT1/STAT3/STAT5 signaling cascade that results in the transcription of IL-5 responsive genes that facilitate activation and survival of eosinophils. Created with biorender.com.

Intereukin-5 (IL-5) is a cytokine released by Th2 cells, group 2 innate lymphoid cells (ILC2), mast cells, and eosinophils [18,49,72,73]. IL-5 is a 50–60 kDa homodimeric glycoprotein that has pro-eosinophilic effects that are orchestrated by binding to the IL-5 receptor (IL-5R) [150]. IL-5R is a heterodimeric receptor complex that consists of IL-5R alpha (CD125) and IL-2Rβ chain (CD122). IL-5R is expressed on eosinophils and basophils (Table 1) [18,72,74]. The beta chain of the receptor is essential for signal transduction, and it is utilized by interleukin-3 (IL-3) and granulocyte-macrophage colony-stimulating factor (GM-CSF). This explains the reason for IL-3 and GM-CSF having the ability to promote the survival of eosinophils [74]. The binding of IL-5 to IL-5R initiates a signaling cascade that results from the dimerization of the receptor signaling chains. This brings JAK1 and JAK2 into proximity to phosphorylate each other. JAK2 is associated with IL-5R alpha (CD125) and JAK1 is associated with IL-2Rβ chain (CD122) [72]. JAK1 and JAK2 phosphorylate the tyrosine kinase associated with the cytoplasmic tail of the cytokine receptor to create a phosphotyrosine docking site for downstream signaling proteins such as signal transducer and activator of transcription (STAT) proteins. STAT1, STAT3, and STAT5 are recruited to the phosphotyrosine docking site, and these STAT proteins become phosphorylated by JAK [72,130]. The phosphorylated STAT proteins dimerize, and the phosphorylated dimerized STAT proteins subsequently dissociate from the cytoplasmic domain to relocate to the nucleus and bind to the STAT-binding site on the DNA to initiate or regulate the transcription of IL-5 responsive genes that facilitate activation and survival of eosinophils (Figure 3) [72,118,119]. Additional downstream signaling proteins that become activated upon being recruited to the phosphotyrosine docking site include phosphoinositide 3-kinase (PI3K) and mitogen-activated protein kinases (MAPK) [72,130]. IL-5 is a cytokine that mediates type 2 immune responses. It is required for the proliferation, maturation, and differentiation of eosinophils and subsequent chemotaxis of eosinophils to the site of allergen-induced inflammation in the conjunctiva [64,72,129,130]. Activated eosinophils produce several mediators including eosinophil peroxidase and eosinophil neurotoxin which are toxic to the ocular surface [49,73]. These toxic substances eventually lead to the breakdown of cellular adhesion and desquamation of epithelial cells of the conjunctiva and cornea [151]. It has been demonstrated that eosinophils also synthesize IL-5 which leads to a cyclical cascade of immunological processes that perpetuate chronic ocular allergic inflammation (Table 2) [152].

### 4.3. Interleukin-6

**Figure 4:**
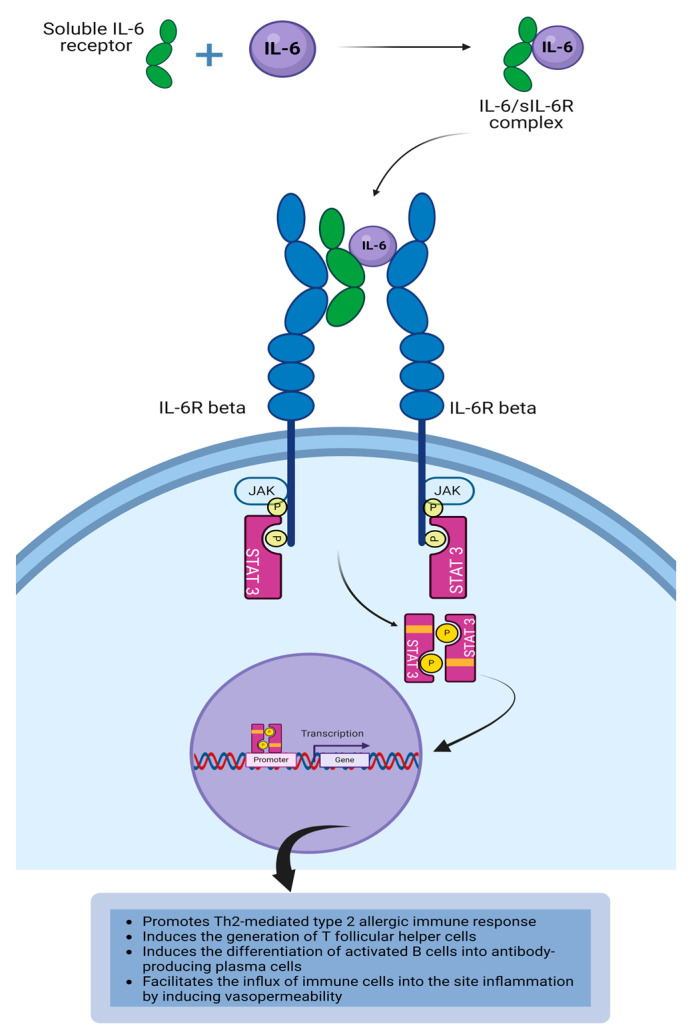
Binding of IL-6/sIL-6R to gp130 results in the IL-6 trans-signaling. This initiates activation of the downstream intracellular signaling cascade via the JAK/STAT3 pathway leading to IL-6-mediated inflammatory responses. Created with biorender.com.

Interleukin-6 (IL-6) is a member of the IL-6 type cytokine family that plays a role in innate and adaptive immune responses [1,2]. IL-6 is produced by dendritic cells, monocytes, macrophages, B cells, epithelial cells, and endothelial cells [3]. It is also produced by T cells, fibroblasts, vascular smooth muscle cells, glial cells, and keratinocytes [4,5]. IL-6 is a pleiotropic cytokine that is involved in anti-inflammatory activity, chronic inflammation, autoimmune disease via autoantibody, vasopermeability, metabolism, and hematopoiesis [6,7]. IL-6 exhibits both anti-inflammatory and pro-inflammatory properties based on the signaling mechanism. Classical IL-6 signaling leads to the generation of an anti-inflammatory process and trans IL-6 signaling results in the induction of pro-inflammatory activities [8]. The IL-6-induced pro-inflammatory activity in type 2 allergic immune responses is mediated by the IL-6 trans-signaling pathway [9,10]. IL-6 receptor (IL-6R) consists of an IL-6R alpha chain and IL-6R beta chain/gp130. The IL-6R alpha chain of the IL-6 receptor is the binding domain for IL-6, whereas the IL-6R beta chain of the receptor mediates signal transduction [10,11]. Membrane-bound IL-6R (CD126) is expressed on lymphocytes, monocytes, macrophages, neutrophils, and hepatocytes (Table 1) [2,11]. It is also expressed on ocular surface epithelial cells and fibroblasts [12]. The binding of IL-6 to mIL-6R leads to the recruitment of 2 gp130 co-receptor molecules (CD130) to form the IL-6R complex [10,13]. Glycoprotein 130 is a highly conserved 130-kDA protein that is widely expressed on many cells [14,15]. Classic IL-6 signaling is associated with the activation of immune cells that express membrane-bound IL-6R (mIL-6R) such as B cells, T cells, neutrophils, macrophages, monocytes, NK cells [16], and ocular surface epithelial cells [12]. The classical signaling via membrane-bound IL-6R mediates an anti-inflammatory response [17]. A disintegrin and metalloproteinase 17 (ADAM17) is a membrane-bound metalloproteinase that mediates the proteolytic cleavage of membrane-bound IL-6R to generate soluble IL-6R (sIL-6R) [17,18]. IL-6 can act on target cells that do not express IL-6R alpha by forming a complex with sIL-6R (IL-6/sIL-6R complex) that binds to and activates gp130 bound to the cell surface to form a high-affinity IL-6R complex. IL-6 binds to the cell membrane IL-6R leading to the recruitment of gp130 that dimerizes to form the IL-6R complex that activates JAK1 and JAK2. This is the classical signaling via IL-6. Soluble IL-6R mediates the trans-signaling pathway when IL-6 binds extracellular soluble IL-6R to form an IL-6/sIL-6R complex that binds to cell surface gp130 to form the IL-6R complex that initiates the downstream signaling cascade [6]. Dimerization of IL-6R and gp130 (IL-6R beta) also brings the JAKs together, which in turn, auto-phosphorylate each other, and activate the tyrosine residues on the cytoplasmic domain of IL-6R beta. The phosphorylated tyrosine residues become phosphotyrosine docking sites for STAT proteins [6,11,15]. The phosphorylation of STAT3 by JAKs occurs when STAT3 binds to the phosphotyrosine docking sites on the cytoplasmic domain of the cytokine receptor [11,15]. The phosphorylated STAT3 homodimers become dimerized and dissociate from the cytokine receptor [11]. These dimerized STAT3s translocate into the nucleus where they bind to the STAT-binding site on the DNA to initiate or regulate the transcription of IL-6-responsive genes that mediate inflammation (Figure 4) [6,11]. IL-6 trans-signaling via IL-6/sIL-6R/gp130 complex promotes pro-inflammatory activity of the cytokine, whereas classic IL-6 signaling via IL-6/mIL-6R/gp130 complex promotes an anti-inflammatory response and activation of the acute phase response [7]. Activation of cells via the IL-6/sIL-6R/gp130 complex is associated with the pro-inflammatory activity of IL-6 [17,18]. As such, IL-6 can activate cells that do not express mIL-6R [9]. Histamine can activate epithelial cells to release IL-6 and upregulate the expression of gp130 on conjunctival epithelial cells. It has been demonstrated that there are elevated levels of gp130 in the tears of patients with allergic conjunctivitis, and as such, gp130 can play a role in allergic conjunctivitis [14]. It has been demonstrated that significant levels of soluble IL-6 receptor produced by proteolytic cleavage of membrane-bound IL-6R were present in the tears of patients with VKC and giant papillary conjunctivitis (GPC). IL-6 trans-signaling could have a role to play in propagating chronic inflammation at the ocular surface [19]. In the setting of an allergen challenge, it has been demonstrated that there is increased IL-6 binding to sIL- 6R to promote Th2-mediated type 2 allergic immune response [3]. It has been demonstrated that IL-6 can increase the proliferation of mast cells, thereby contributing to the immunopathology associated with mast cell-mediated diseases such as allergic conjunctivitis [15]. IL-6 is a 26 kilodalton (kDa) secreted protein that possesses pleiotropic functions in both innate and adaptive immune responses. In the context of innate immunity, IL-6 plays a pro-inflammatory role at the site of infection with the generation of acute phase proteins from hepatocytes [11]. In the context of adaptive immunity, IL-6 in synergy with TGF-beta and IL-23 drives the differentiation of activated CD4^+^T cells into Th17 cells [11,20]. IL-6 is essential for the development of the germinal center, as it drives the differentiation of activated CD4^+^T cells into T follicular helper cells that are required for providing signals to B cells in the germinal center to produce high-affinity antibodies [5,6,11]. IL-6 can activate vascular endothelial cells to induce endothelial dysfunction and vascular permeability as well as the recruitment of immune cells and molecules to the site of inflammation [4,11]. IL-6 can induce the proliferation of mast cells, thereby contributing to the immunopathology associated with mast cell-mediated diseases such as allergic conjunctivitis (Table 2) [15].

### 4.4. Interleukin-9

**Figure 5:**
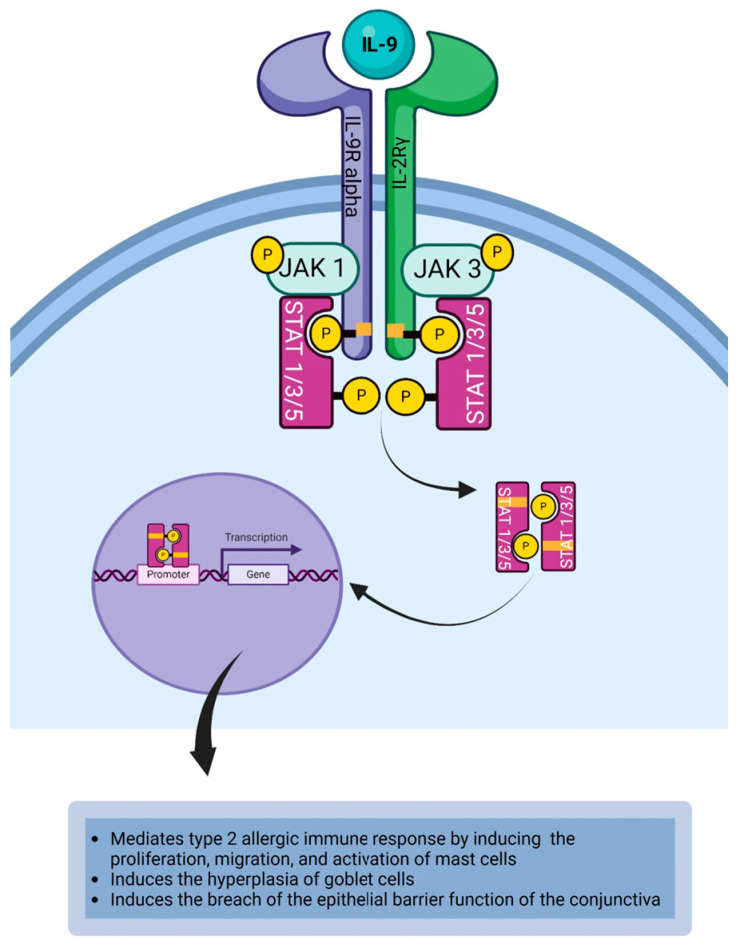
IL-9 binds to the IL-9R complex to initiate the JAK1/JAK3/STAT1/STAT3/STAT5 signal cascade that results in generating IL-9-mediated inflammatory response in the conjunctiva. Created with biorender.com.

IL-9 is a pleiotropic cytokine produced by Th9 cells [15], mast cells [81], and eosinophils [82]. Th2 cells and basophils are other cellular sources of IL-9 [83]. IL-9 is a 14 kDa glycoprotein that belongs to the four-helix bundle cytokine family [153]. IL-9 receptors (IL-9R) are expressed on epithelial cells, fibroblasts, granulocytes, lymphocytes, macrophages, and mast cells [84,85]. This receptor is also reported to be expressed on T cells, smooth muscle cells [54], and B cells [83]. IL-9 binds to and activates the IL-9 heterodimeric receptor complex (CD129) that consists of IL-9R alpha chain and IL-2Rγ chain (CD132) (Table 1) [84]. The interaction between IL-9 and IL-9R complex results in signal transduction where the cytoplasmic domain of the receptor becomes activated and dimerized. This results in the activation of JAK1 on the IL-9R alpha and activation of JAK3 on the IL-2Rγ chain as well as the cross-phosphorylation of JAK1 and JAK3. The activated JAKs phosphorylate the tyrosine kinases on the cytoplasmic domain of the receptor to create a phosphotyrosine docking site for STAT proteins such as STAT1, STAT3, and STAT5 [15,54]. The recruited STAT proteins are phosphorylated by the JAK to form dimers. Each recruited STAT protein binds to the SH2 domain of the other STAT protein to form a phosphorylated STAT dimer, which subsequently dissociates from the cytoplasmic domain of the cytokine receptor to relocate to the nucleus and where they bind to the STAT-binding site on the DNA to initiate or regulate the transcription of IL-9 responsive genes that mediate the response of the cytokine (Figure 5) [118,119]. IL-9 is a T cell and mast cell growth factor that plays a role in the disease process of allergic inflammation [83]. It is of note that mast cells are the major targets of IL-9 produced by Th9 cells during allergic inflammation [83,154]. IL-9 signaling via its receptor expressed on mast cells induces the activation, proliferation, and migration of mast cells during allergen challenge [84]. Sismanopoulos et al. demonstrated that IL-9 can induce mast cells to secrete vascular endothelial growth factor (VEGF) [132]. IL-9 induces the secretion of chemokine from epithelial cells and promotes the hyperplasia of goblet cells [83] located in the conjunctival layer. Interaction of IL-9 with its receptor on eosinophils induces the upregulation of IL-5R alpha chain expression on eosinophils [133]. Gounni et al. [133] have demonstrated that eosinophils secrete IL-9 which enhances the production of IgE-secreting plasma cells from activated B cells. In the murine model of allergic experimental conjunctivitis, Hu et al. [136] demonstrated that the IL-9/IL-9R pathway was associated with barrier dysfunction of ocular epithelial cells. A breach of the epithelial barrier function of the conjunctiva could facilitate the access of allergens on the ocular surface to the antigen-presenting cells and mast cells in the conjunctival stroma, thereby contributing to the pathophysiology of allergic conjunctivitis. The effect of IL-9 on epithelial cells of the conjunctiva continues to be an area of active research. Thus, the IL-9/IL-9R pathway contributes to the inflammatory process in allergic conjunctivitis (Table 2) [136].

### 4.5. Interleukin-10

**Figure 6:**
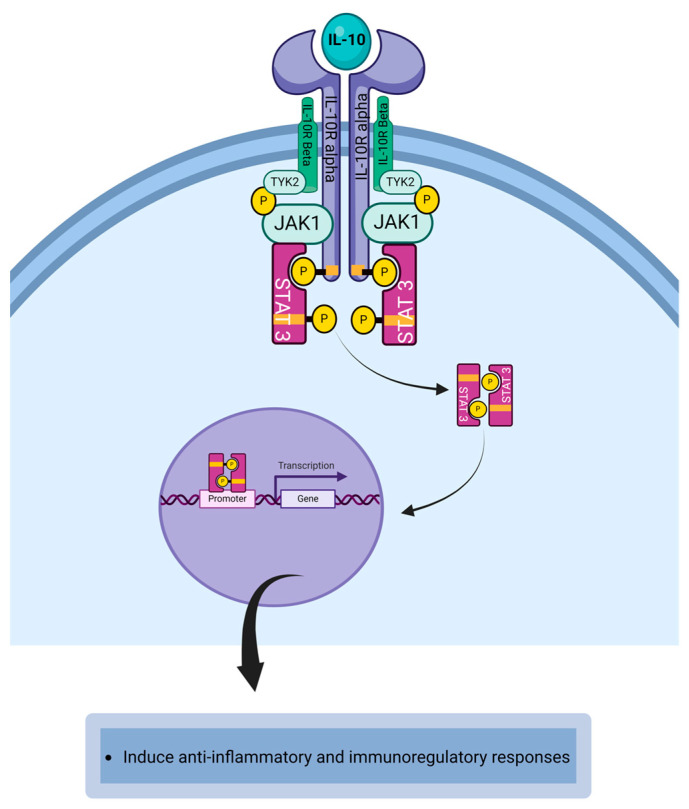
The binding of IL-10 to IL-10R complex initiates the activation of JAK1/TYK2/STAT3 signaling cascade that leads to the development of STAT3-mediated anti-inflammatory responses. Created with biorender.com.

Interleukin-10 (IL-10) is an immunosuppressive cytokine and a member of the class II cytokine family. It is a 37 kDa protein with potent anti-inflammatory properties [21]. IL-10 downregulates class II MHC expression by macrophages and monocytes [22]. IL-10 is secreted by macrophages, monocytes, dendritic cells, neutrophils, mast cells, eosinophils, NK cells, CD4^+^T cells, CD8^+^T cells, and B cells, microglia, and epithelial cells [22,23,24]. IL-10 is released when Th2 cells release cytokines that mediate type 2 allergic immune responses [22,25,26]. In allergic conjunctivitis, IL-10 downregulates the allergic immune response by inhibiting the effector function of immune cells and mediators that participate in type 2 immune-mediated activity [25]. The IL-10-mediated anti-inflammatory response is inversely correlated with the severity of the disease [20]. The IL-10 receptor consists of two IL-10 receptor alpha chains that serve as the binding chain for IL-10 and two IL-10 receptor beta chains that serve as the signal transduction chain [27]. IL-10R is expressed on immune cells, fibroblasts, and epithelial cells (Table 1) [28]. IL-10 binds to the IL-10R alpha chain resulting in the dimerization of IL-10R alpha chain and IL-10R beta chain to form the IL-10 receptor complex [27]. The dimerization of IL-10R alpha and IL-10R beta activates the receptor-associated JAK1 and protein tyrosine kinase 2. JAK1 is associated with IL-10R alpha and TYK2 is associated with IL-10R beta [22,27]. The phosphorylated JAK1 and TYK2 activate the tyrosine residues on the cytoplasmic domain of the IL-10R alpha chain to create a phosphotyrosine docking site for STATs [22,25]. The recruited STAT3 homodimers are phosphorylated by JAK1 and TYK2 and subsequently become dimerized [22]. The dimerized STAT3 dissociates from the cytokine receptor and translocates to the nucleus to bind the STAT-binding site to initiate the transcription of IL-10 responsive genes to induce anti-inflammatory and immunoregulatory responses (Figure 6) [22,25,29]. IL-10 exhibits pleiotropic effects in mediating immunoregulating and suppressing immune reactions during Th2-mediated allergic immune response. IL-10 can reduce the allergen-induced inflammatory response at the conjunctiva [27]. Exposure of IL-10 to allergen-activated dendritic cells can promote the generation of allergen-specific regulatory T cell that suppresses Th2-mediated inflammation in the conjunctiva exposed to allergen [27,30]. IL-10 secreted by regulatory T cells (Tregs) can inhibit the expression of FcεRI on mast cells, thereby preventing the degranulation of mast cells [20].

### 4.6. Interleukin-13

**Figure 7:**
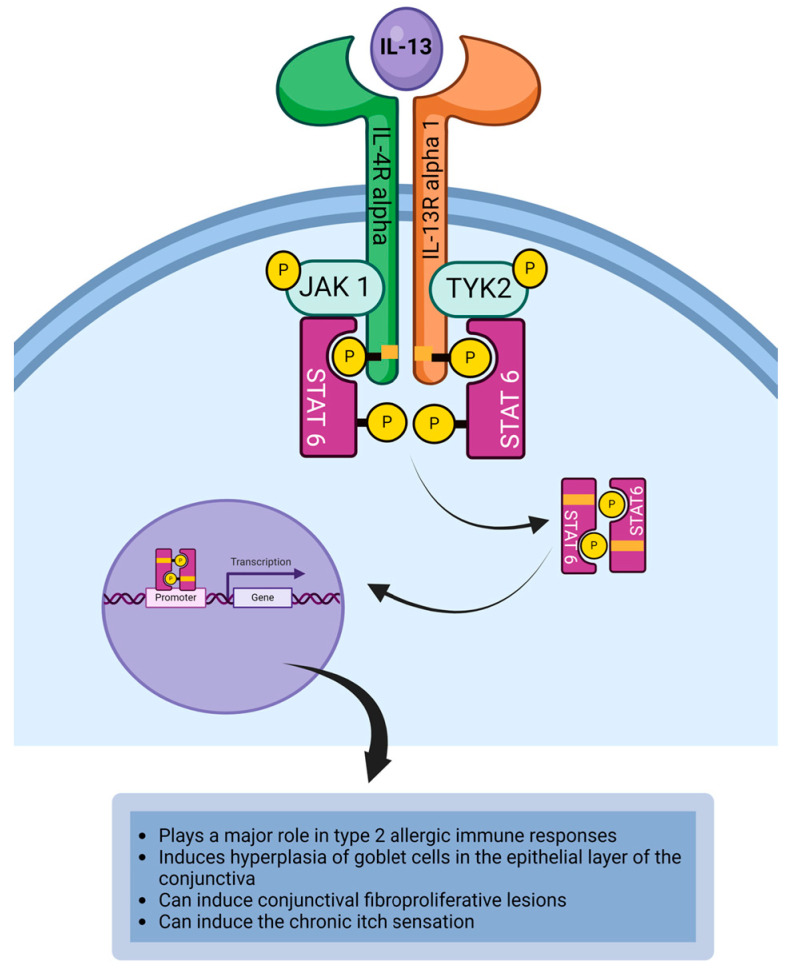
The binding of IL-13 to the receptor induces the JAK1/TYK2/STAT6 signaling cascade that results in generating IL-13-mediated allergic immune response in the conjunctiva. Created with biorender.com.

Interleukin-13 (IL-13) is a pleiotropic cytokine with similar functions to IL-4. It is secreted by Th2 cells, mast cells, type 2 innate lymphoid cells (ILC2), and basophils [91,92]. It plays a significant role in the pathophysiology of type 2 immune responses in the conjunctiva exposed to allergens. IL-13 is another four-helix bundle cytokine. Its function overlaps with IL-4 given that they share a common receptor, IL-4R alpha [155]. IL-13 receptor (IL-13R) is a heterodimeric receptor complex that is made up of IL-4R alpha chain and IL-13R alpha1 chain. IL-13R is expressed on smooth muscle cells, fibroblasts, keratinocytes, and goblet cells (Table 1) [49,91,92]. The binding of IL-13 to the receptor induces the signaling cascade that results in the dimerization of the receptor. This brings JAK1 and TYK2 into proximity to phosphorylate each other. JAK1 is associated with IL-4R alpha chain and TYK2 is associated with IL-13R alpha1 chain. The phosphorylated JAKs activate the tyrosine residues on the cytoplasmic domain of the IL-13R to create a phosphotyrosine docking site for STAT6 [91,92,115,156,157,158]. The JAKs bind and activate the STAT6, and the phosphorylated STAT6 forms a phosphorylated STAT dimer, which subsequently dissociates from the cytoplasmic domain of the receptor to relocate to the nucleus. In the nucleus, phosphorylated STAT dimers bind to the STAT-binding site on the DNA to initiate or regulate the transcription of IL-13 responsive genes required for mediating the response of the cytokine (Figure 7) [68,69,118,119]. Overexpression of IL-13 is linked to the development of pathophysiological features consistent with allergic conjunctivitis including the hyperplasia of goblet cells in the epithelial layer of the conjunctiva [14,137]. It is of note that mucoid discharge does occur in allergic conjunctivitis due to IL-13-induced hypersecretion of mucin from activated goblet cells [48,138,139]. Additionally, IL-13 acts on epithelial cells and smooth muscle cells to promote allergic inflammation [18]. Interaction of IL-13 with its receptor expressed on vascular endothelial cells induces the upregulation of VCAM-1 to induce vasopermeability that facilitates the adhesion and subsequent extravasation of eosinophils and T cells to the site of allergic inflammation in the conjunctiva [157,158]. Receptors for IL-13 are expressed by mast cells and binding of IL-13 to its receptors on mast cells is associated with mast cell activation and subsequent promotion of allergic inflammation (Table 2) [123,124,159].

### 4.7. Interleukin-25

**Figure 8:**
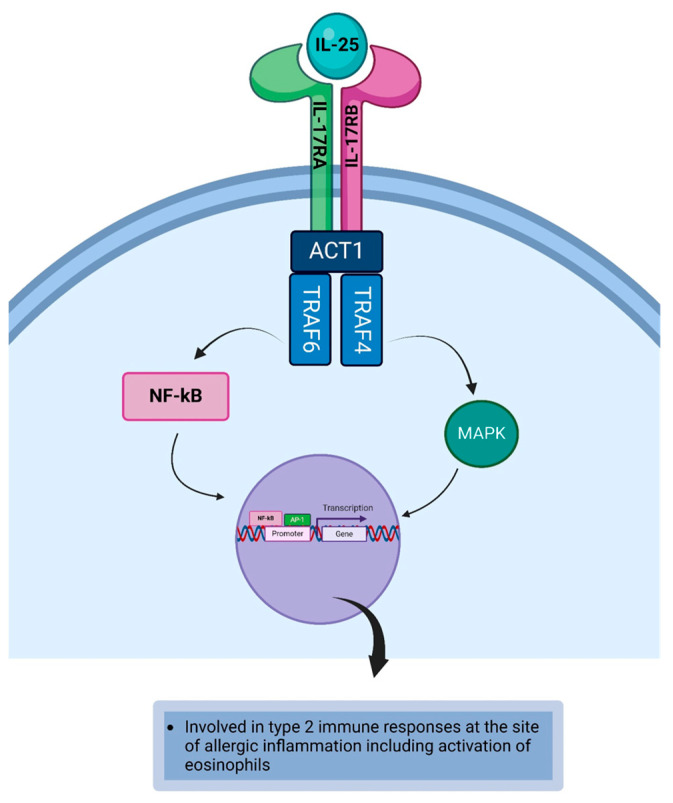
The binding of IL-25 to its receptor induces the transcription of IL-25 responsive genes via the TRAF6/NFκB-dependent signaling cascade and TRAF4/MAPK/AP-1-dependent signaling pathway. Created with biorender.com.

Interleukin-25 (IL-25) is a member of the IL-17 cytokine family [160]. IL-25, also known as IL-17E, is a hydrophobic peptide coded by a gene located on chromosome 14 [161]. IL-25 is derived from Th2 cells, mast cells [16], basophils, eosinophils [93], epithelial cells [94], and endothelial cells [95]. IL-25 receptor (IL-25R), a heterodimeric receptor complex that consists of IL-17RA and IL-17RB, is expressed on eosinophils [96] and T cells (Table 1) [97]. The cytoplasmic SER/IL-17R domain (SEFIR domain) is expressed on IL-17R, whereas Toll/IL-1R-like loop (TIR-like loop, TILL) domain and CCAAT/enhancer binding protein beta activation domain (C/EBPβ–activation domain/CBAD) is expressed on IL-17RA [140,162,163,164]. The binding of IL-25 to its receptor induces the SEFIR domain to recruit and activate nuclear factor kappa B Activator 1 (Act1). The activated Act1 is an adapter protein that recruits and binds TNF receptor-associated factor 6 (TRAF6). The activated TRAF6 activates nuclear factor kappa B (NFκB), which migrates to the nucleus and binds to the specific DNA target to initiate gene transcription for encoding proteins that induce the response mediated by the cytokine. Thus, IL-25 promotes Act1/TRAF6/NFκB-dependent signaling cascade to induce Th2-mediated immune responses [164,165]. Additionally, Act1 binds and activates TRAF4, and the activated TRAF4 recruits E3 ligase smadubiquitin regulatory factor 2 (SMURF2). TRAF4-SMURF2 mediates the degradation of Deleted in Azoospermia-associated protein 2 (DAZAP2) to initiate the activation of tyrosine residues Y444 and Y454 on the IL-17RB by JAK2, leading to the creation of a phosphotyrosine docking site. The recruited STAT5 is activated by JAK2 [140,166,167] and this is followed by the dissociation and translocation of the phosphorylated STAT5 dimer to the nucleus, where it binds to transcription regulatory proteins to form a transcription regulatory complex. The transcription regulatory complex binds to the DNA target to initiate gene transcription for encoding proteins that mediate the response of the cytokine (Figure 8) [118,119,168]. The activated TRAF4 activates mitogen-activated protein kinase (MAPK), which subsequently activates the activator protein-1 (AP-1) transcription factor that migrates to the nucleus and binds to the specific DNA target. This will initiate the transcription of protein-coding genes required for the cytokine-induced type 2 allergic immune responses [140]. IL-25 has the potential to stimulate angiogenesis at the site of allergic inflammation via the upregulation of vascular endothelial growth factor (VEGF) and VEGF receptor on endothelial cells of the blood vessels [141] in the conjunctiva. Additionally, IL-25 can act on fibroblasts to promote the generation of VEGF and VEGF receptor [141]. Angkasekwinai et al. reported overexpression of IL-25 by lung epithelial cells in allergy transgenic mice [94]. While this epithelial cell-derived cytokine continues to be investigated, it is believed that epithelial cells of the conjunctiva can express IL-25 which leads to allergic inflammation of the ocular surface [169]. IL-25 can activate eosinophils during the allergic immune response, and as such, IL-25 promotes type 2 allergic immune responses (Table 2) [142].

### 4.8. Interleukin-31

**Figure 9:**
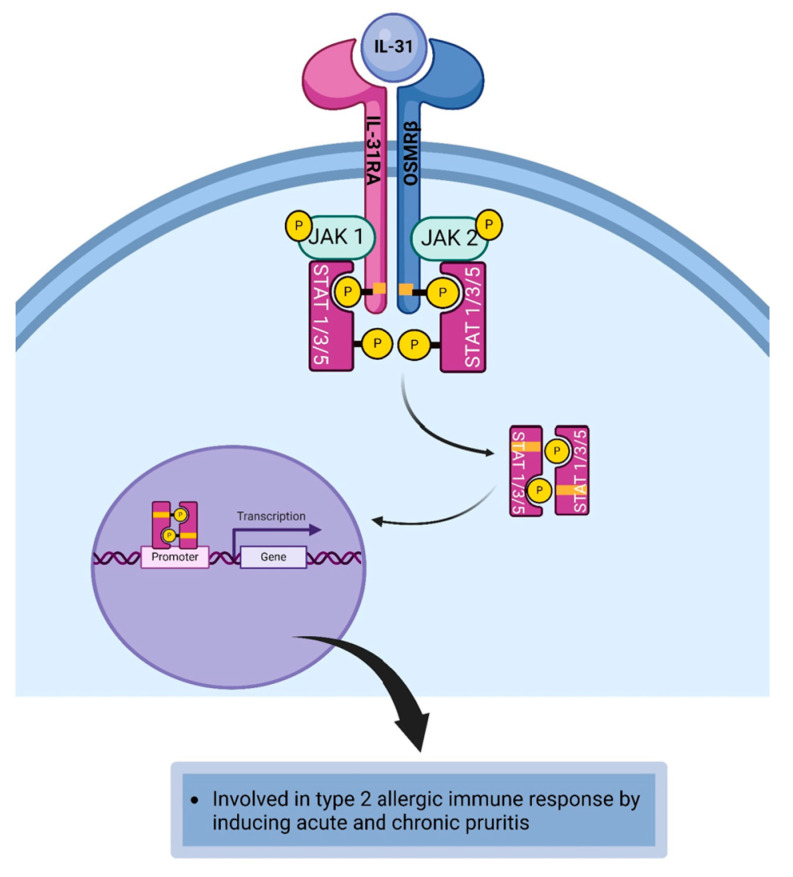
The interaction between IL-31 and its receptor results in the activation of JAK1/JAK2/STAT1/STAT3/STAT5 signal cascade that results in generating IL-31-mediated pathophysiological features of type 2 allergic immune responses. Created with biorender.com.

Interleukin-31 (IL-31) is a pleiotropic cytokine that plays a key role in the pathogenesis of the itch sensation [170]. IL-31 has a four-helix bundle structure encoded by a gene located on chromosome 12q24.31 [17]. IL-31, a member of the IL-6 family of cytokines, is secreted by Th2 cells, mast cells, eosinophils, basophils, monocytes, and dendritic cells [17,98,99]. IL-31 is responsible for inducing the itch sensation at the site of inflammation during allergic immune responses [171,172]. The IL-31 receptor (IL-31R) is a heterodimeric receptor complex that is made up of a gp-130-like receptor chain IL-31RA and oncostatin M receptor beta (OSMRβ) subunit [100]. IL-31 has a pleiotropic physiological role in allergic diseases because of its ability to bind to its receptor expressed on immune and nonimmune cells [17]. IL-31R is expressed on dendritic cells, eosinophils, mast cells, basophils, sensory neurons, and monocytes (Table 1) [98,100,101]. The interaction between IL-31 and its receptor results in the activation of three downstream signaling pathways, which include the JAK/STAT pathway, phosphatidylinositol 3-kinase/protein kinase B (PI3K/Akt) pathway, mitogen-associated protein kinase (MAPK) pathway [17,98,170]. In the JAK/STAT pathway, binding of IL-31 to the IL-31R triggers the dimerization of the receptor signaling chains. This brings JAK1 and JAK2 into proximity to phosphorylate each other. The JAKs phosphorylate the tyrosine residues on the cytoplasmic domain of the cytokine receptor to create a phosphotyrosine docking site for STAT proteins. STAT1, STAT3, and STAT5 are recruited to the docking sites and become activated and dimerized upon phosphorylation by JAKs [17,170]. The phosphorylated dimerized STAT proteins dissociate from the cytoplasmic domain of the cytokine receptor and relocate to the nucleus where they bind to STAT-binding sites on the DNA to initiate or regulate the transcription of IL-31 responsive genes required for the IL-31-induced pathophysiological changes in type 2 allergic immune responses (Figure 9) [118,119]. IL-31 overexpression has been demonstrated in patients experiencing itching [173,174]. It is important to note that the itchy sensation experienced by individuals with allergic conjunctivitis is regulated by the somatosensory neurons located in the trigeminal ganglion [175,176]. It has been revealed that IL-31 mediates the acute and chronic itch sensation, and the binding of IL-31 to its receptor expressed on sensory nerve fiber in the conjunctiva initiates this itch sensation [100]. It is of note that histamine is associated with acute itch sensation [120]. IL-31 can interact with its receptor on mast cells and eosinophils to induce type 2 mediated inflammation, whereas IL-31 engages IL-31R expressed on sensory nerve fibers to induce the activation of transient receptor potential vanilloid 1 (TRPVI) and transient receptor potential ankyrin 1 (TRPA1) to transmit signals that cause the itch sensation [98,177]. Thus, one of the major pathophysiological roles of IL-31 in allergic eye disease is inducing pruritus following the binding of the cytokine to its receptor expressed on sensory neurons in the conjunctiva (Table 2) [17].

### 4.9. Interleukin-33

**Figure 10:**
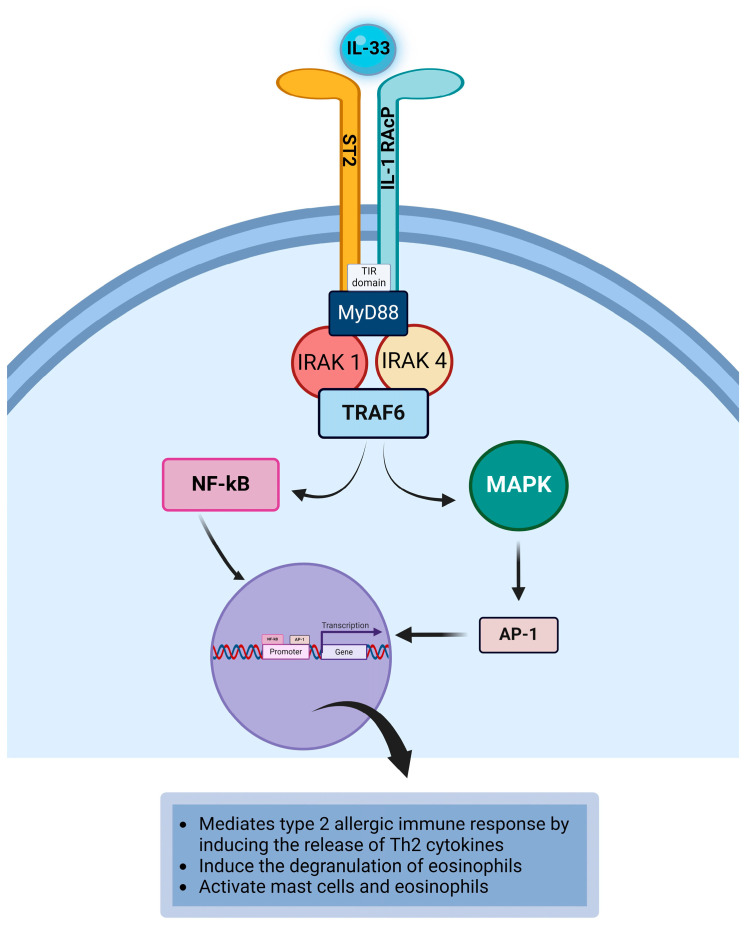
The binding of IL-33 to its receptor induces the MyD88/IRAK1/IRAK4/TRAF6/NFκB- dependent signaling pathway and the TRAF6/MAPK/AP-1-dependent signaling pathway. This initiates the transcription of IL-33-responsive genes that are responsible for the development of the pathophysiological changes observed in allergic conjunctivitis. Created with biorender.com.

Interleukin-33 (IL-33) is a member of the IL-1 family of cytokines that participates in both innate and adaptive immune responses, particularly in type 2 immune response, via its action on mast cells and Th2 cells [105]. IL-33 is a 31 kDa secreted glycoprotein that possesses proinflammatory effects [178]. IL-33 has a pathophysiological role in allergic conjunctivitis [54] because it promotes the allergen-induced inflammatory process in the conjunctiva observed in individuals with allergic conjunctivitis [179]. The cellular source of IL-33 includes endothelial cells, fibroblasts, epithelial cells, mast cells, and smooth muscle cells [18,102,103,104]. IL-33 receptor consists of the primary receptor suppression of tumorigenicity 2 (ST2) and the coreceptor IL-1 receptor accessory protein (IL-1RAcP) [103,105,106]. Dendritic cells, mast cells, Th2 cells, Th9 cells, fibroblasts, macrophages, basophils, epithelial cells, endothelial cells, and eosinophils express ST2, and as such, these cells are targets of IL-33 (Table 1) [103,106,107,108]. Binding of IL-33 to its receptor leads to the bringing together of ST2 and IL1RacP, and the dimerization of the Toll/Interleukin-1 receptor (TIR) domain of the receptor. Myeloid differentiation factor 88 (MyD88), a TIR domain binding protein, is recruited to the TIR domain. Interleukin-1 receptor-associated kinase (IRAK) proteins are also recruited and activated by MyD88. IRAK1 and IRAK4 mediate the activation of tumor necrosis factor acceptor associated factor 6 (TRAF6) [103,104,180,181]. TRAF6 activates the transcription factor nuclear factor kappa B (NFκB), which relocates to the nucleus and binds to the specific DNA target to initiate gene transcription for encoding proteins that induce the response mediated by the cytokine. Additionally, TRAF6 induces the phosphorylation of mitogen-activated protein kinases (MAPK) leading to the activation of the transcription factor AP-1. The activated AP-1 relocates to the nucleus and binds to the specific DNA target to initiate the transcription of IL-33 responsive genes required to induce the response mediated by the cytokine (Figure 10) [104,106,180,182]. IL-33 is considered an alarmin because it is released during inflammation and when there is tissue damage. Tissue damage and/or mechanical irritation of epithelial cells and endothelial cells induce these cells to secrete IL-33. IL-33 can exacerbate the inflammation of the conjunctiva upon re-exposure of the conjunctival epithelial cells to allergens [103]. IL-33 promotes type 2 immune responses in allergic conjunctivitis by mediating the release of Th2 cytokines by Th2 cells and mast cells [54]. Iikura et al. [183] demonstrated that IL-33 could enhance the effector function of mast cells in allergic disorders in the presence or absence of costimulation of mast cells via allergen-induced crosslinking of IgE-FcεRI on primed mast cells [183] to promote the release of cytokines and chemokines [103]. Because mast cells express IL-33R, IL-33 can participate in mast cell-mediated pathophysiological changes observed in allergic conjunctivitis via the induction of cytokines and chemokines released from mast cells [69,183,184]. IL-33 can induce the degranulation of eosinophils to release eosinophilic mediators that are toxic to the ocular surface [103]. Cherry et al. [143] demonstrated that IL-33 could bind to its receptor expressed on eosinophils to induce eosinophil-mediated inflammation during allergic responses. As such, IL-33 can activate mast cells and eosinophils during type 2 immune responses in allergic diseases [54,143]. In a mouse model of allergic conjunctivitis, Matsuba-Kitamura demonstrated that conjunctival epithelial cells release IL-33 [185]. Their results suggest that IL-33, which contributes to the activation of Th2, mast cells, and eosinophil, can be a therapeutic target for treating individuals with allergic conjunctivitis (Table 2).

### 4.10. Thymic Stromal Lymphopoietin

**Figure 11:**
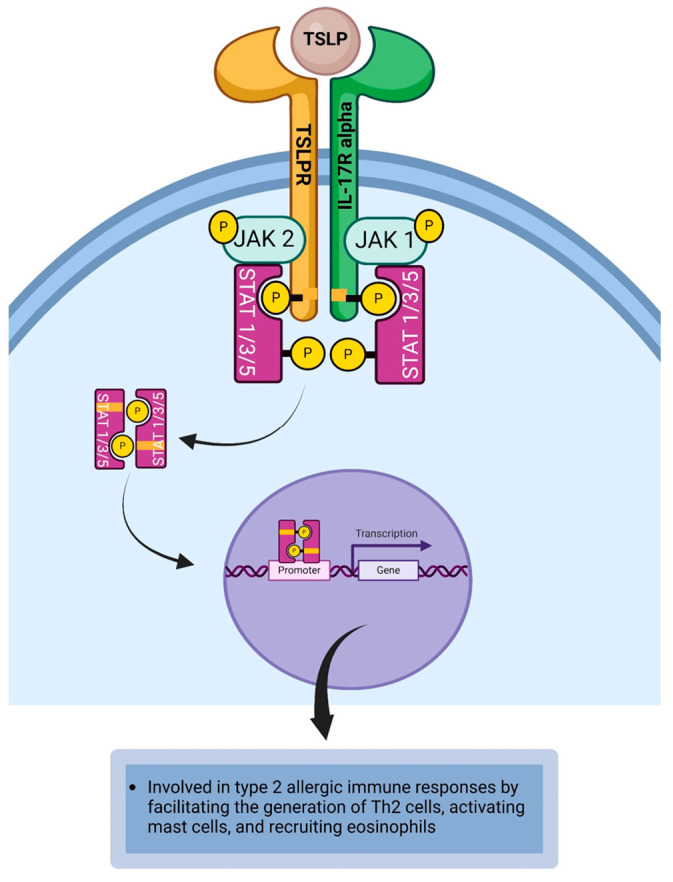
TSLP Signaling Cascade: TSLP binds to the TSLPR complex to induce the JAK1/JAK2/STAT1/STAT3/STAT5 signaling cascade that results in the transcription of TSLP-responsive genes that exacerbates the inflammatory process associated with Th2-mediated immune responses. Created with biorender.com.

Thymic stromal lymphopoietin (TSLP) is an IL-7-related cytokine that is expressed by epithelial cells, fibroblasts, mast cells, and smooth muscle cells [54,109]. TSLP is a polypeptide with four alpha helices held together by disulfide bonds [186]. The TSLP receptor (TSLPR) complex consists of an IL-7R alpha chain and a TSLPR chain [54,110,111,112]. Dendritic cells, mast cells, T cells, basophils, neurons, and eosinophils express receptors for TSLP (Table 1) [54,113]. TSLP binds to the TSLPR complex to induce the signaling cascade that results in the dimerization of the receptor. This brings JAK1 and JAK2 into proximity to cross-phosphorylate each other and activate the tyrosine residues on the cytoplasmic domain of the receptor to create a phosphotyrosine docking site for STAT proteins. JAK1 is associated with the IL-7R alpha chain and JAK2 is associated with the TSLPR chain. STAT1, STAT3, and STAT5 are recruited and then bind to JAKs leading to the phosphorylation of STATs by JAKs [54,113,187,188,189,190]. The phosphorylated STATs form phosphorylated STAT dimers, which subsequently dissociate from the cytoplasmic domain of the receptor to relocate to the nucleus and bind to STAT-binding site [189,190,191] on the DNA to initiate or regulate the transcription of TSLP responsive genes required for mediating the pro-inflammatory response of the cytokine (Figure 11) [118,119]. Utilizing a case-control design, Zheng et al. examined conjunctival scrapings and tears from eighty subjects (of which 20 were controls) with different clinical forms of allergic conjunctivitis. They found that TLSP levels were significantly elevated in subjects with allergic conjunctivitis compared to the controls [192]. Following the expression of TLSP on the ocular mucosal surface, it binds to TSLP-receptors (TSLPR) expressed by cells implicated in the inflammatory process. Studies with animal models [111,145] and on humans [144] have revealed that TSLPR is predominantly found on resident dendritic cells. The binding of TSLP to TSLPR expressed on dendritic cells leads to the activated dendritic cells upregulating the expression of CD40, CD80, CD86, and OX40L [193,194,195]. The TSLP-stimulated dendritic cell facilitates the activation of naïve CD4^+^T cells to proliferate and differentiate into Th2 cells that secrete IL-4, IL-5, IL-9, IL-13, IL-25, and IL-31. Thus, TSLP has a role to play in allergic conjunctivitis and allergic keratoconjunctivitis [144,145]. TSLP can activate mast cells to secrete IL-13, a cytokine that plays a pathophysiological role in allergic conjunctivitis [109,146]. TSLP-stimulated dendritic cells can release chemokines such as CCL11 and CCL17 [54,113] that recruit eosinophils and Th2 cells to the site of allergic inflammation, respectively [18,109,147]. TSLP can exacerbate the inflammatory process associated with Th2-mediated immune responses by producing IL-4, IL5, IL-9, and IL-13. IL-4 and IL-13 induce allergen-specific B cells to proliferate and differentiate into IgE-secreting plasma cells. IL-5 recruits eosinophils to the site of allergic inflammation, whereas IL-9 activates mast cells that secrete IL-4 and IL-13 [109,113,196]. TSLP engages TSLPR expressed on sensory nerve fibers to induce the activation of transient receptor potential ankyrin 1 (TRPA1) to transmit signals that cause pruritus (Table 2) [148,149].

## 5. Cytokines as Therapeutic Targets in the Management of Allergic Conjunctivitis

Most cytokines involved in type 2 immune responses are also implicated in the pathophysiology of allergic conjunctivitis. Therefore, these cytokines can be targeted as novel immunotherapeutic modalities either by preventing their release or counteracting their effects through competitive and noncompetitive antagonism of their receptors borne by target cells. Such modalities could be optimized for managing both acute and chronic forms of allergic conjunctivitis. IL-4, IL-5, IL-6, IL-9, IL-13, IL-31, and TSLP mediate type 2 immune responses during allergic inflammation via the JAK/STAT pathway [197]. As such, inhibiting JAKs associated with the receptor of these cytokines can attenuate the pathophysiological changes mediated by these cytokines. Tofacitinib is an inhibitor of JAK1, JAK2, and JAK3. It has been shown to attenuate the clinical expression of allergic conjunctivitis in mouse models of allergic conjunctivitis (Table 3) [131,198]. Abrocitinib, an oral JAK1 inhibitor, that curtails the signaling of IL-4 and IL-13, has been reported to provide early itch symptom relief in the atopic dermatitis (Table 3) [199,200]. The development of an ophthalmic-formulated version of this drug will serve to eliminate or minimize the involvement of IL-4 and IL-13 in mediating type 2 inflammatory processes in allergic conjunctivitis. Such medication has the potential for the management of acute and chronic symptoms of ocular allergy. Targeting IL-4R alpha, a component shared by both IL-4R and IL-13R, can attenuate the type 2 inflammatory response mediated by IL-4 and IL-13, respectively [201]. Dupilumab is a subcutaneously injected monoclonal antibody that blocks IL-4R alpha and is currently utilized in the treatment of atopic dermatitis (Table 3) [202]. Considering the similarities between the role of IL-4 in the pathophysiology of atopic dermatitis and allergic conjunctivitis, the utility of Dupilumab in the management of the latter disease is worth exploring. As such, monoclonal antibody against IL-4R alpha can attenuate the IL-4 and IL-13-induced pathophysiological changes in allergic conjunctivitis. Utilized in the management of asthma, Mepolizumab and Benralizumab, are monoclonal antibodies that target IL-5 and IL-5Rα, respectively (Table 3) [203]. A major outcome of using these biologics is the attenuation of the recruitment of eosinophils to the site of allergic inflammation. This medication may have huge potential in the management of chronic ocular allergy, where mediators released by eosinophils cause inflammation and damage to the ocular surface. Targeting IL-5 is a good immunotherapeutic modality to ameliorate the damage and remodeling of the ocular surface due to the toxic effects of mediators released by eosinophils. Thus, monoclonal antibody against IL-5R alpha can inhibit eosinophilic inflammation in the conjunctiva [204]. Tocilizumab is a monoclonal antibody against IL-6R including sIL-6R and IL-6/sIL-6R complex (Table 3) [205] and it has been approved for the treatment of rheumatoid arthritis and cytokine release syndrome [206]. Tofacitinib can block IL-6 signaling because of its ability to inhibit JAK1 and JAK2 [131]. Sgp130Fc (Olamkicept) consists of the extracellular portion of human gp130 and the constant domain of human IgG1 antibody. Sgp130Fc-mediated blockade of IL-6 trans-signaling ameliorates the inflammatory response associated with IL-6/sIL-6R/gp130 complex on target cells [207]. Tocilizumab blocks both IL-6 classical signaling and IL-6 trans-signaling, whereas Sgp130Fc is an inhibitor of only IL-6 trans-signaling (Table 3) [208]. The blockade of IL-6 can ameliorate the immune and pathophysiological role of IL-6 in type 2 allergic immune responses [15]. IL-9 can induce the synthesis of IgE, increase mucus production from goblet cells, and mediate the accumulation and proliferation of mast cells at the site of allergic inflammation. As such, blocking the interaction of this cytokine with its receptor will potentially inhibit IL-9-mediated goblet cell hyperplasia and activation of mast cells [153] in the conjunctiva. Using a mouse model of allergic asthma, Ballantyne and colleagues demonstrated that blockade of IL-25 leads to the reduction in clinical expression due to type 2 cytokine-induced allergic inflammation [209]. Because of the involvement of IL-25 in the pathophysiology of allergic conjunctivitis, targeting IL-25 or IL-25R on effector cells is a potential modality for attenuating IL-25 mediated type 2 immune response [162]. IL-31 is a cytokine that causes itching and breach of the epithelial barrier function. It has been shown that Nemolizumab, a humanized monoclonal antibody against the IL-31RA chain, was effective at attenuating the itch sensation induced by IL-31 (Table 3) [210,211]. Monoclonal antibodies targeting IL-31 and its receptor could be developed as a potential antipruritic therapy with the clinical therapeutic benefits of attenuating the itch sensation associated with allergic eye disease. In a murine model, Matsuba-Kitamura demonstrated that conjunctival epithelial cells release IL-33, a cytokine that promotes the type 2 inflammatory process in the eye. They suggested that this cytokine can exacerbate allergic conjunctivitis due to the ability of the cytokine to induce the generation of Th2-derived cytokines from Th2 cells and mast cells [185]. Tozorakimab is a human anti-IL-33 monoclonal antibody that inhibits IL-33 mediated type 2-mediated immune response due to allergen-induced inflammation (Table 3) [212]. This finding suggests that IL-33 can be a therapeutic target with broad effects in treating individuals with allergic conjunctivitis. We previously highlighted the central role of TSLP in mediating mucosal inflammatory diseases of which ocular surface allergy and asthma are prime examples. In the phase 3 trial, Tezepelumab, a monoclonal antibody targeting TSLP, significantly minimized exacerbation of symptoms across all seasons in patients with uncontrolled asthma, including those with seasonal and perennial allergies (Table 3) [213]. This therapy offers a promising therapeutic alternative in the treatment of seasonal and perennial allergic conjunctivitis.

## 6. Conclusions

The discussion in this review has highlighted the role of Th2- and Th9-derived cytokines in inducing pathophysiological changes in the ocular surface of patients with allergic conjunctivitis. These cytokines interact with their receptors expressed on nonimmune and immune cells in the conjunctiva to induce immunological and pathological changes along with the resultant clinical manifestations in allergic conjunctivitis.

In summary, IL-4, produced by different immune cells in the conjunctiva, plays a versatile role in exacerbating allergic inflammatory responses and is responsible for clinical signs such as conjunctival hyperemia, chemosis, and papillae. IL-5 mediates the involvement of eosinophils, which, in turn, produce toxic mediators that damage the ocular surface. IL-6 plays a role in facilitating the proliferation of mast cells during the allergic response. IL-9 promotes the growth and proliferation of mast cells and T cells. It also activates conjunctival epithelial cells to release chemokines as well as induce goblet cell hyperplasia. IL-13 functions similarly to IL-4. IL-25 is known to facilitate the development of type 2 allergic inflammatory responses. IL-31 mediates both acute and chronic itch sensations, with the cytokine binding to its receptor on sensory nerve fibers in the conjunctiva. IL-33 activates mast cells and eosinophils which results in their degranulation, a process that is crucial for exacerbating allergic reactions on the ocular surface. TSLP is one of the cytokines that has its receptors expressed by several immune cells, as such its involvement in initiating and moderating an ongoing inflammatory process in an allergic ocular surface disease cannot be understated.

An improved understanding of the role of these cytokines in the pathophysiology of allergic conjunctivitis as well as the cellular and molecular mechanisms involved in such pathophysiological changes is critical in developing therapeutic agents that block the signaling cascade associated with the interaction of these cytokines with their receptors. Thus, blocking cytokines from interacting with their receptors has the potential to provide insight into identifying therapeutic modalities including immunopharmacology that target cytokine–cytokine receptor interaction with the therapeutic intent of ameliorating these pathophysiological changes associated with allergic conjunctivitis. Further research is necessary to fully elucidate the therapeutic benefits of cytokine-targeted therapy in allergic conjunctivitis.

## Figures and Tables

**Table 1:** Cellular sources and cytokine receptors expressed by immune and nonimmune cells.

Cytokines	Cellular Sources	Cytokine Receptors	Cytokine Receptor Components	Cells Expressing Cytokine Receptors
**IL-4**	Th2 cells, mast cells, eosinophils, and basophils [65,66].	IL-4R	IL-4R alpha (CD124), IL-2Rγ chain (CD132) [68,69].	Monocytes, macrophages, fibroblasts, T cells, smooth muscle cells, and B cells [54].
**IL-5**	Th2 cells, group 2 innate lymphoid cells (ILC2), mast cells, and eosinophils [18,49,72,73].	IL-5R	IL-5R alpha (CD125), IL-2Rβ chain (CD122) [18,72,74].	Eosinophils and basophils [18,72,74].
**IL-6**	Dendritic cells, monocytes, macrophages, B cells, epithelial cells, and endothelial cells [75]. T cells, fibroblasts, vascular smooth muscle cells, glial cells, and keratinocytes [76,77].	IL-6R	IL-6R alpha chain (CD126) and IL-6R beta chain/gp130 (CD130) [78,79,80].	Lymphocytes, monocytes, macrophages, neutrophils, and hepatocytes [79,80].
**IL-9**	Th9 cells [15], mast cells [81] and eosinophils [82]. Th2 cells, eosinophils, and basophils [83].	IL-9R	IL-9R alpha chain, IL-2Rγ chain (CD132) [84].	Epithelial cells, fibroblasts, granulocytes, lymphocytes, macrophages, mast cells [84,85], T cells, smooth muscle cells [54], and B cells [83].
**IL-10**	Macrophages, monocytes, dendritic cells, neutrophils, mast cells, eosinophils, NK cells, CD4+T cells, CD8+T cells, and B cells, microglia, and epithelial cells [86,87,88].	IL-10R	IL-10R alpha chain and IL-10R beta chain [89].	Immune cells, fibroblasts, and epithelial cells [90].
**IL-13**	Th2 cells, mast cells, type 2 innate lymphoid cells (ILC2), and basophils [91,92].	IL-13R	IL-4R alpha chain, IL-13R alpha1 chain [49,91,92].	Smooth muscle cells, epithelial cells, fibroblasts, B cells, macrophages, endothelial cells, and goblet cells [49,91,92].
**IL-25**	Th2 cells, mast cells [16], basophils, eosinophils [93], epithelial cells [94], and endothelial cells [95].	IL-25R	IL-17RA, IL-17RB [96,97].	Eosinophils [96] and T cells [97].
**IL-31**	Th2 cells, mast cells, eosinophils, basophils, monocytes, and dendritic cells [17,98,99].	1L-31R	IL-31RA, oncostatin M receptor beta (OSMRβ) subunit [100].	Dendritic cells, eosinophils, mast cells, basophils, sensory neurons, and monocytes [98,100,101].
**IL-33**	Endothelial cells, fibroblasts, epithelial cells, mast cells, and smooth muscle cells [18,102,103,104].	1L-33R	Suppression of tumorigenicity 2 (ST2), IL-1 receptor accessory protein (IL-1RAcP) [103,105,106].	Dendritic cells, mast cells, Th2 cells, Th9 cells, fibroblasts, macrophages, basophils, epithelial cells, endothelial cells, and eosinophils [103,106,107,108].
**TSLP**	Epithelial cells, fibroblasts, mast cells, and smooth muscle cells [54,109].	TSLPR	IL-7R alpha chain, TSLP receptor chain [54,110,111,112].	Dendritic cells, mast cells, T cells, basophils, sensory neurons, and eosinophils [54,113].

**Table 2:** Pathophysiological roles of cytokines.

*Cytokines*	*Pathophysiological Role of Cytokines*
IL-4	IL-4 and IL-13 can induce the chronic itch sensation via the IL-4R alpha-JAK1 pathway [120]. IL-4 can stimulate conjunctival fibroblast to proliferate and produce excess collagen leading to the development of papillae on the palpebral conjunctiva [128]. IL-4 induces the production of IgE-secreting plasma cells [122]. IL-4 induces induce vasodilation and vasopermeability by upregulating the expression of vascular cell adhesion molecule 1 (VCAM-1) on endothelial cells of blood vessels [126,127].
IL-5	IL-5 induces the proliferation, maturation, differentiation, and chemotaxis of eosinophils to the site of allergen-induced inflammation in the conjunctiva [64,72,129,130].
IL-6	IL-6 is essential for the development of the germinal center, as it drives the differentiation of activated CD4+T cell into T follicular helper cells that are required for providing signals to B cells in the germinal center to produce high affinity antibodies [77,79,131]. IL-6 can activate vascular endothelial cells to induce endothelial dysfunction and vascular permeability as well as the recruitment of immune cells and molecules to the site of inflammation [76,79].
IL-9	IL-9 induces the proliferation, migration, and activation of mast cells during allergic immune responses [84]. IL-9 can induce mast cells to secrete vascular endothelial growth factor (VEGF) [132]. IL-9 induces the secretion of chemokine from epithelial cells [83]. IL-9 induces the hyperplasia of goblet cells [83]. IL-9 induces the upregulation of IL-5R alpha chain on eosinophils [133]. IL-9 facilitates the production of IgE-secreting plasma cells [134,135]. IL-9 can induce the breach of the epithelial barrier function of the conjunctiva [136].
IL-13	IL-13 induces hyperplasia of goblet cells in the epithelial layer of the conjunctiva [14,137]. IL-13 induces the hypersecretion of mucin from activated goblet cells [48,138,139]. IL-13 can activate sensory neurons to induce the chronic pruritus [120]. IL-13 can promote the development of papillae on the palpebral conjunctiva by inducing the proliferation of conjunctival fibroblasts [47].
IL-25	IL-25 initiates and exacerbates type 2 immune responses at the site of allergic inflammation [140]. IL-25 has the potential to stimulate angiogenesis at the site of allergic inflammation [141]. IL-25 can activate eosinophils during the allergic immune response [142].
IL-31	IL-31 mediates the acute and chronic itch sensation [100].
IL-33	IL-33 promotes type 2 immune responses in allergic conjunctivitis by mediating the release of Th2 cytokines by Th2 cells and mast cells [54]. IL-33 can induce the degranulation of eosinophils to release eosinophilic mediators that are toxic to the ocular surface [103]. IL-33 can activate mast cells and eosinophils during type 2 immune responses in allergic diseases [54,143].
TSLP	TSLP-stimulated dendritic cells facilitate the generation of Th2 cells [144,145]. TSLP activates mast cells to secrete mediators that promote type 2 allergic immune responses in the conjunctiva [109,146]. TSLP-stimulated dendritic cell can release chemokines that recruit eosinophils and Th2 cells to the site of allergic inflammation in the conjunctiva [18,109,147]. TSLP activates sensory nerve fibers to induce pruritus [148,149]

**Table 3:** Summary of therapeutic drugs targeting cytokines.

Therapeutic drug	Targeted Cytokine	Mechanism
**Tofacitinib**	IL-4, IL-6 and IL-13	JAK1, JAK2 and JAK3 inhibitor [131,198].
**Abrocitinib**	IL-4 and IL-13	JAK1 inhibitor [199,200].
**Dupilumab**	IL-4 and IL-13	IL-4R alpha antagonist [202].
**Benralizumab**	IL-5	IL-5R alpha antagonist [203].
**Mepolizumab**	IL-5	Inhibits IL-5 release [203].
**Tocilizumab**	IL-6	Blocks IL-6R [205]
**Olamkicept**	IL-6	Blocks IL-6 trans-signaling [208].
**Nemolizumab**	IL-31	IL-31RA chain antagonist [210,211].
**Tozorakimab**	IL-33	Inhibits IL-33 signaling [212].
**Tezepelumab**	TSLP	Blocks TLSP [213].

## Data Availability

No new data were created or analyzed in this study. Data sharing does not apply to this article.
